# Risk and protective factors of relapse in patients with first-episode schizophrenia from perspectives of health professionals: a qualitative study in northeastern Thailand

**DOI:** 10.12688/f1000research.53317.1

**Published:** 2021-06-25

**Authors:** Jarunee Intharit, Khanogwan Kittiwattanagul, Wisit Chaveepojnkamjorn, Kukiat Tudpor

**Affiliations:** 1 Faculty of Public Health, Mahasarakham University, Kantarawichai, Maha Sarakham, 44150, Thailand; 2 Khon Kaen Rajanagarindra Psychiatric Hospital, Mueang, Khon Kaen, 40000, Thailand; 3 Department of Epidemiology, Faculty of Public Health, Mahidol University, Rajthevee, Bangkok, 10400, Thailand

**Keywords:** first-episode schizophrenia, qualitative research, relapse, substance abuse, caregivers

## Abstract

**Background:  **Schizophrenia is a serious mental illness that can relapse after treatments. Risk and protective factors for relapse are dependent on multicultural contexts.

**Objective:** To identify risk and protective factors related to relapse in first-episode schizophrenia (FES) in northeastern Thailand from perspectives of health professionals.

**Methods:** This qualitative research collected data from 21 health professional staff members (psychiatric nurses, psychiatrists, psychologists, social workers, occupational therapists and nutritionist) of a tertiary psychiatric hospital of northeastern Thailand who had been involved in mental health care for schizophrenia for at least 5 years by in-depth interviews and group interview using semi-structured interview schedule. Content analyses was used to identify staff perception of factors that put patients at risk of relapse.

**Results: **Data analyses demonstrated that factors related to relapse in FES patients were drug adherence (drug discontinuation, limited access to new generation drugs, self-dose reduction and skipping medication, and poor insight), family factors (stressful circumstances and family supports), substance abuses (narcotics, addictive substances, caffeinated drinks), concurrent medical illness (insomnia, thyroid diseases, and pregnancy-related hormonal changes), and natural course of disease.

**Conclusion:** Factors affecting relapse in FES was not only drug adherence. Family factors, drug abuses, and concurrent health status should be also taken into account. A comprehensive mental health care program should be developed for FES patients in the region.

## Introduction

Schizophrenia is a chronic mental health disorder manifested by positive symptoms (suspiciousness, hallucinations, delusions, impaired cognitive ability, and disorganized speech or behavior) and negative symptoms (impairments in attention, loss of volition, social withdrawal, poverty of speech, and affective flattening) ^1, 2^. The prevalence of the disease varies between sites with median of approximately 15/100,000 persons ^3^. The initial treatments of inpatient schizophrenia patients are antipsychotic drugs and other complementary interventions such as psychosocial therapy (social skill training and cognitive behavior therapy) and medical nutrition therapy ^4– 6^. Even though schizophrenic patients with first-episode psychosis (FEP) can be completely recovered by treatment programs, relapse can still occur. Relapse during the first few years after onset of the FEP is an important determinant for long-term clinical and functional outcome ^7^. Rate of relapse in the first two years after the FEP is 28–43% ^8^. The FEP constitutes a substantial burden for families, society, and health-care systems around the world ^9– 11^. Risk and protective factors in symptom severity of schizophrenia are different among countries and cultures ^12^. Hence, identification of modifiable risk factors that could influence relapse is a crucial for planning treatment strategies. Therefore, the present qualitative study was aimed to investigate risk and protective factors in patients with first-episode schizophrenia in northeastern Thailand from perspectives of health professionals.

## Methods

### Study design and participants

This descriptive qualitative study based on Caelli’s generic principles using in-depth semi-structured interviews and focus group of health professionals to identify the risk and protective factors for relapse in patients with first-episode schizophrenia ^13^. The health professionals participating in the study were purposively sampled from Khon Kaen Rajanagarindra Psychiatric Hospital (n = 21; male = 4, female = 17; aged between 34 and 52 years old) based on their qualification of at least three-year work experiences in the psychiatric hospital and informed consent. The study was carried out between January and July 2018. The research report complies with consensus-based minimum reporting guidelines for qualitative research (COREQ) (a copy of the checklist can be found in the *Reporting guidelines*). Data can be found at https://osf.io/4fhe5/?view_only=11d3b735eee44c61ac8e77b8bc15986a.

Researcher study profiles and information about prior relationship/contact with research participants can be found in the *Extended data* ^14^.

### Ethical approval

All procedures have been approved by the Ethical Review Committee for Human Research, Faculty of Public Health, Mahasarakham University (PH010/2560) and were carried out in accordance with the ethical principles in the Declaration of Helsinki. Clinical research methodology has been approved by the Thai Clinical Trials Registry (TCTR) TCTR20190923001. Pseudonyms were used to protect identity. Written informed consent forms were obtained from all participants and a copy of the form can be found in the *Extended data* ^14^.

### Procedures

An experienced interviewer (J.I.), unknown to the participants and not involved in their work processes, conducted face-to-face and group semi-structured interviews allowing them reflecting points of view on the risk and protective factors. The interviews consisted of two parts – demographic data and 16 items of interviews on problems and causes of mental health status, severity of problems, perceived impact of problems, and needs for mental health care. The content validity of all the items has been confirmed by index of item-objective congruence (IOC). Only the items with IOC scores ≥ 0.75 were qualified and used. The interviews were undertaken and audio-recorded in Khon Kaen Rajanagarindra Psychiatric Hospital consulting rooms for approximately 90 minutes; and transcribed verbatim by the interviewer. Field notes were also made.

### Data analysis

Data analysis was ongoing during the data collection. Transcripts were translated to English and coded using an agreed scheme by three independent coders (authors J.I., K.K. and K.T.). The transcripts were then sorted and organized using NVivo® 11 software package for managing and analyzing qualitative research data (QSR International, Melbourne, Australia). The data were coded and categorized to conceptualize emerging themes highlighted by the patients. All the answers from the participants were transported to the software, and the relevant sections from each questionnaire were coded and put into different categories. Using concept maps, the coded categories were further grouped to derive a particular theme from each group. Most representative quotations from the patients were used to bolster the themes. The transcripts were returned to the participants for correction proof. Coding tree analysis can be found in the *Extended data* ^14^.

## Results

###  Demographic data of participants

Twenty-one health professionals (four males and seventeen females) were psychiatrists (5), psychiatric nurses (10), psychologists (2) social workers (2), a nutritionist, and an occupational therapist with mean age of 44.5±6 years old and average duration of services of 22.4±8.7 years. Fifteen staff work in the outpatient department and six persons serviced in the inpatient department. All details are shown in Table 1.

**Table 1.** Demographic data of the health professionals.

Characteristics	Percentage (n) or Mean±SD	Range
Gender (male/female)	19 (4)/81 (17)	
Age (year)	44.5±6	34–52
Length of career (year)	22.4±8.7	6–38
Job position		
Psychiatrist	5 (23.8)	
Psychiatric nurse	10 (47.6)	
Psychologist	2 (9.5)	
Social worker	2 (9.5)	
Nutritionist	1 (4.8)	
Occupational therapist	1 (4.8)	
Education level		
Bachelor degree	5 (23.8)	
Higher than bachelor degree	16 (76.2)	
Department		
Outpatient	15 (71.4)	
Inpatient	6 (28.6)	

### Risk and protective factors

Based on an analysis with the software, risk and protective factors were categorized into five main themes: drug adherence, family factors, substance abuses, concurrent medical illness, and natural course of disease as summarized in Table 2.

**Table 2.** Themes and subthemes constructed from the semi-structured interviews of the health professionals.

No.	Theme	Subtheme
1	Drug adherence	1.1 Drug discontinuation
		1.2 Limited access to new generation drugs
		1.3 Self-dose reduction and skipping medication
		1.4 Poor insight
2	Family factors	2.1 Stressful circumstances
		2.2 Family supports
3	Substance abuses	
4	Concurrent medical illness	
5	Natural course of disease	

### Theme 1: Drug adherence

Adherence is not limited to patient’s compliance to the treatment plan, but also their proper behavioral changes after diagnosis and life style adaptation in accordance with the treatment plan of the medical staff. Failure to follow medical prescription was attribute to various reasons, which could be further subdivided into four subthemes as follows.

***Subtheme 1.1: Drug discontinuation***

Patients took antipsychotic drugs until they felt better. They thought that they were fully recovered and hence skipped or discontinued taking drugs. Other reasons were unawareness of importance of the drugs, forgetfulness, and fear of side effects. Medical staff’s opinions are quoted as follows.

Psychiatrist 2: “From my experience, relapse can be due to skipping drugs. The symptoms used to be controllable recur. In some cases, the relapse lasts for 1 week, some may last for 6–12 months.”

Psychiatrist 3: “After we’ve sent them back home, some patients do not take drugs, causing relapse.”

Psychiatrist 6: “Drug discontinuation, yes, many cases.”

Community psychiatric nurse 1: “I used to follow up cases by phone. Some patients said that they had been completely recovered. No more drugs needed. This is one reason why they had to return to the hospital.”

Some health professional provided reasons for discontinuation.

Psychiatric nurse 1: “The patient said the drugs made him drooling and difficult speaking.”

Psychiatric nurse 2: “Some antipsychotic drugs have side effects like poor or rigid walking.”

Psychiatric nurse 3: “Refraining from taking drugs during the day since it made him drowsy and unable to teach (the patient was a teacher). Office workers tended to adjust dose of the drug themselves and didn’t want to take it due to the side effects like bad image and personality.”

***Subtheme 1.2: Limited access to new generation drugs***

High-quality and costly new-generation antipsychotic drugs were not prescribed for patients under basic healthcare coverage scheme. Therefore, these drugs were not accessible for economically poor people.

Psychiatrist 4: “It’s about side effects. Some drugs have many side effects. Patients can’t tolerate and have to quit. For example, intramuscular injection of first-generation long-acting drugs, which are on the list (of the drugs for basic healthcare coverage scheme) can cause EPS (extrapyramidal side effects) more easily…rigid and stiff body. Meanwhile, less side effects can be seen in the patients using drugs like Paliperidone palmitate. But this drug is not covered by the scheme, very expensive, and so not accessible by most of the patients. Because of the side effects, patients were lost from follow-up or even from the whole treatment program and finally relapsed.”

Psychiatrist 3: “Drugs play a big role. There’s big difference between typical and atypical drugs. The atypical ones prevent relapse better. So, patients who cannot access atypical drugs lose opportunities for better treatments.”

Psychiatrist 2: “Patients have no access to atypical drugs according to their healthcare scheme…limited only to two atypical drugs. Another atypical drug is very expensive and not in the list (the National List of Essential Medicines of Thailand), so we cannot prescribe.”

Psychiatrist 3: “At one point, when patients don’t respond to drugs, not so many choices left for us. Only typical drugs are in the list, so we lose control…no other choices. This makes it more complicated and more expensive for treatment cost. In some hospitals, they have just only first-generation antipsychotic drugs for newly diagnosed schizophrenia patients.”

***Subtheme 1.3: Self-dose reduction and skipping medication***

Patients took drugs until symptoms subsided and thought they fully had recovered. In some patients, they were worried about side effects. Then they arbitrarily decided to reduce drug dosage as stated below.

Psychiatrist 6: “Patients think they get better, so they adjust drug dosage. They’re worried if they have taken them too much.”

Community psychiatric nurse 1: “In some cases, they always skip drugs due to side effects. Instead of coming back for dose adjustment, they do it themselves or simply quit it. So, recurrence is so severe that they have to be re-admitted.”

***Subtheme 1.4: Poor insight***

Some patients lacked ability to recognize psychotic symptoms as illness. Thus, they did not need any treatments as mentioned below.

Community psychiatric nurse 2: “In my point of view, a frequently found barrier to mental health care was that majority of patients denied their presence of illness…this was very clear. Then we forced them to come for treatment until they recovered. But they discontinued drugs when they were back home.”

Occupational therapist: “First thing, they denied their illness. Then, yes, they received drugs, but not taking them.”

Psychologist 2: “It’s a consequence from their denial of illness and kind of not understanding. They got home, not taking drugs, becoming relapsed, and coming back here again.”

In contrast, patients who well cooperated with care team would have better health in long term, being more peaceful or recovered from schizophrenia symptoms. They knew their needs for continuous treatments, managed to take drugs on their own. So, they came to receive medication for continuous treatments.

Psychologist 1: “It’s compliance with treatments. For schizophrenia, drug adherence is important anyway. Continuous use of drugs has a huge effect. We have been seeing some cases routinely come for checkup, years past and still coming to receive new drugs.”

Community psychiatric nurse 2: “From my experience, patients wouldn’t get relapse if they found jobs and had enough income. Meanwhile, medical staff took care of providing drugs, both oral and injection drugs. Community health staff gave mental support.”

Community psychiatric nurse 2: “This case had first-episode schizophrenia with a problem of illness denial. So, we had to motivate his recognition of illness. This would guide him how he should take care for himself.”

### Theme 2: Family factors

Family factors in schizophrenia patients including stressful circumstances and family supports were addressed as causes of pressure as stated below.

***Subtheme 2.1: Stressful circumstances***

Persons with schizophrenia might not be able to appropriately cope with stressful circumstances such as losing their important persons, poor relationships, aggressive expressions, and poor communication.

Psychiatrist 2: “From my experience, there are 4–5 reasons for relapse…the fourth one is stress. They might encounter life crisis like family loss and couldn’t cope with it. As a consequence, relapse happened despite they were taking drugs.”

Psychiatrist 2: “Stress is one of relapse aggravating factors, especially the stress from critical lost events in their lives. When they lost, they failed to conform to the situation. Even though medications were continued, relapse could still occur.” 

Community nurse 2: “The factor that leads to severe first-episode and relapse, from my experience, is not alcohol, but principally is family pressure. When patients had family problems, they resorted to using alcohol and tobacco. Good friends would support their mind, but bad friends would ask them for drinking to release pressure. The drinking party is a talking panel for pressure.”

Psychologist 1: “Well, a high expressed emotion has a high impact no matter how smart patients are. It can be very hostile as a cause of accumulative stress. The high expressed emotion is the variable that worsens psychotic symptoms in most cases.”

Psychiatric nurse 1: “Some patients had been spoiled since their childhood, not accepting any rational talks. If they failed to control themselves, then the problems resumed. In some cases; on the other hand, if their parents were overly critical, psychotic symptoms might relapse. The patients could feel annoyed and lose self-control.”

Social worker 2: “Family problems like finance and debt can cause relapse and hospitalization. Particularly, poor communication among family members on financial topics can be stressful and aggravate the symptoms. In many cases, patients mentioned mother’s complaints of their laziness. Then they responded with ironic drinking of alcohol and smoking.”

***Subtheme 2.2: Family supports***

Most of schizophrenia patients receive family caring and supports ^15^. These caregivers have been involved from the beginning – observing changed behaviors, bringing them to the hospitals, and supporting their routine cares in accordance with treatment plan until the patients can live their lives in society ^16^. Therefore, competency of the caregivers is importance for them. In this present study, five subtopics related to family supports were found i.e., absenteeism of caregivers, unskilled caregivers, caregiver’s illiteracy on schizophrenia, family’s attitude, and poverty. When patients live alone or live with ever-busy family members, they have to confront with absenteeism of a primary caregiver. They lack continuous support, making it difficult to follow treatment plans as stated below.

Community psychiatric nurse 1: “In community, some family members were so overloaded that they lost contact with the hospital. Well, it’s a matter of family income. In this case, a patient had to live alone and discontinued medications as he thought he had been fully recovered.”

Psychiatric nurse 1: “The major problem was that patients lived with old grandparents. Other family members were tied up. The patients didn’t know when they should take antipsychotic drugs or when they had to come back for prescription refill. In some families, primary caregiver never showed up.”

Educated caregivers play important roles in supporting and encouraging patients to follow treatment plans until they fully recover, having no relapse, and live their normal lives in society ^17^. In this present study, it was found that some caregivers had inadequate skills to be in charge of drug adherence, hospital appointment, narcotic drug avoidance as well as guidance for patient’s self-improvement. Examples of the statements are presented as follows.

Community psychiatric nurse 2: “Look, what has actually brought them to alcohol and other substances? Really, it’s their families. Lack of family support and caring can cause relapse. This is the key factor.” 

Community psychiatric nurse 2: “It’s just like a chain reaction. If they received good family supports, they would have stopped thinking about alcohol, ending a triggering process. In contrast, if their families were unsupportive, they would lose their spiritual anchors. This would eventually worsen their symptoms.”

Community psychiatric nurse 1: “In fact, there’s problem in community. Most of caregivers for psychiatric patients are older persons.”

Psychiatric nurse 1: “The primary caregiver was old. When she was busy, a neighbor would bring a patient to the hospital and back home. Then the neighbor went back to work. At home, the patient denied taking medicines. Then the caregiver couldn’t control the patient, not competent to give a care.”

Psychologist 2: “For the first-episode patients, their family members still wouldn’t know how to take care of them. We might educate them on mental health issues. At first, it might not be clear for them. When the patient was calm and able to sleep well, symptoms seemed to be improving. The caregivers tended not to strictly instruct the patients. Even they instructed, the patients might not obey. Then the caregivers couldn’t do anything. On occasion, they were just powerless to control their mentally ill children.”

Lack of knowledge on etiology, symptoms, treatments, healthcare, and relapse prevention among family members and caregivers was another important risk factor as mentioned here.

Psychiatric nurse 1: “In the first-episode patients, family members tended not to understand and didn’t believe that it’s schizophrenia. They thought it’s all about superstition.”

Psychologist 1: “Family’s understanding of treatment process and related factors was important. Weak parents wouldn’t be able to control their children and lacked confidence to take care. If patients drank, the parents dared not dissuade them and had no idea what to do. Sometimes the parents were stress factors themselves – blaming of laziness without understanding the symptoms.”

Social worker 2: “In their point of view, family’s members didn’t realize that alcohols could aggravate the symptoms.”

Social worker 2: “Family’s members didn’t know how to manage patient’s behaviors. So, they were afraid that they might too strictly control the patient and situation would be getting worse. When they realized that the patients were mentally ill, the parents tended to treat them like physically ill persons. Some parents wouldn’t let their mentally ill children help with any housework, worrying about imposing too much stress on them. They still had to learn how to take care for the patients. Let them do some activities.”

In contrast, if caregivers were well-informed and adequately knowledgeable about schizophrenia, they would be able to support patients as addressed below.

Psychologist 1: “If family members understood, it would reduce a lot of risk factors. They should understand process of treatments and had emotional maturity.”

Community psychiatric nurse 2: “These factors were interrelated. If the family support was good, the patient would stop drinking alcohols and think about the family. Contrarily, if the family was bad, the patient would be without someone to lean on; and in turn the symptoms could be worsened.”

In the beginning, family members tended to have negative feeling toward mental illness such as disgrace and denial. The patients would not collaborate and the family didn’t not bring them to treatments as exemplified below.

Psychiatric nurse 1: “Most of the patients never said they were ill.”

Social worker 1: “Some patients and their families didn’t accept an illness. They thought the patients were just normal and it’s too late when they arrived at the hospital. There’re two types of the families – protecting and denying. They tried to protect the patients or deny their illness as they’re afraid of social stigma.

Psychologist 1: “In some cases, their families had negative attitude. They considered the patients as the weak persons. This attitude diminished the self-worth of persons. So, we had to support the patients, not only blaming them.”

Psychiatric nurse 2: “In some community, people stigmatized patients. They tried to relegate the patients from the community.”

In contrast, if patient’s family members had positive attitude and accepted the illness, they would escort the patients to the hospital as well as encourage and support them as stated below.

Psychiatrist 4: “As of lately, attitude of families toward psychiatric patients was better than in the past. Mental illness was previously seen as stigma, but it’s now more accepted as illness. In the past, some patients were confined and enchained, but not anymore. They are now allowed to live together with other family members and provided with medicines. They are not blamed as troubles, but considered as the ill persons, who need treatments and cares.”

Psychiatrist 2: “I think that nowadays society has been wider open. In the past, psychiatric patients were chained and secretly kept in a storeroom. Lately, people in community accept and collaborate to help prevent them from relapse and let them be parts of the community. In some community, the patients have a chance to participate in charity or work for a little extra money. This reflects societal appreciation of the patients. As members of the community, they can get some money from a village fund to pay for taxi cabs, which bring them here to the hospital.”

Some of patients and their families were poor, lived in a distance from the city, and lacked facilities, making it difficult for them to come for continuous follow up as shown in the following statements.

Psychiatrist 3: “Psychosocial factor was that they lived so far away in the backcountry with limited basic needs and living costs; thus, coming to the hospital was difficult for them.”

Psychiatric nurse 1: “Some family members said they couldn’t come, no money. Some had to wait for monthly subsistence allowance from the government fund to pay for a traveling cost. So, we asked them for an appointment only once. Then our doctors would prescribe drugs they could take at a local primary care center.”

Psychiatric nurse 2: “Economic status could indirectly affect the patient’s symptoms. Most of the cases were poor and ill, having no money to come when the symptoms became worse. Caregivers were worried about work and had no time to bring the patients to the hospital.”

Psychiatric nurse 1: “Most of the caregivers were old and poor. For example, they didn’t even have telephones. Then they gave us neighbor’s phone numbers, but turned out to be the wrong ones. It’s difficult for them to access online communication.”

### Theme 3: Substance abuse

Some patients used narcotic drugs such as methamphetamines (meth pills and crystal meth), alcohols, cigarettes, energy drinks, coffee, and weight loss medications unwarily of their psychotic effects. The health professionals shared their experiences as narrated below. 

Psychiatrist 2: “From my experience, there were 4–5 reasons of relapse. One of these was substance use. It was an important cause of changing psychotic symptoms. The most frequently found were psychoactive substances like energy drinks, alcohols, or narcotics. Rice whisky and beer were mainly used by men. Meanwhile, diet pills were used by women to counteract weight gain, a side effect of antipsychotic drugs. Frequently, they quit the antipsychotic drugs and used diet pills and diet coffee drinks, especially in female teenagers.”

Psychiatric nurse 1: “We had problem with readmission due to intractable factors. For example, liquor stores were easily accessed, patients were unaware of their own symptoms, and how they could manage the symptoms.”

Social worker 2: “Relapse in some cases resulted from alcohol drinking regardless of meth pill use. Alcohol is the psychoactive substance. Some patients said they didn’t drink whisky, but only energy drink. In fact, the one they talked about was an alcoholic energy drink. In some female patients, they believed that herbal liqueurs were good for health, which was not true. Actually, the herbal liqueurs could cause relapse. Other issues were cigarette smoking, and coffee.”

Psychiatric nurse 5: “Some first-episode schizophrenia cases had been treated for 3–4 months until fully recovered. Then they got back to work for a couple of years, drinking alcohols. Then it became a long story of relapse afterwards.”

Psychiatric nurse 1: “The thing was they quit medications, resorting to alcohol and drugs. As a result, relapse happened so severely like self-destruction and assaulting others. Some used energy drinks, coffee powder (not the drinking one), white whisky, and meth pills.” 

Psychiatric nurse 2: “Beside antipsychotic drugs, a second problems were addictive substances, most frequently alcohol or even underestimated substances like coffee and energy drinks. These were considered as triggering factors of relapse.”

### Theme 4: Concurrent medical illness

The patients could have relapse due to other health problems, for example, sleeplessness, illness, pregnancy, and other conditions with medications as stated below.

Psychiatrist 2: “Sleeplessness. If patients did not sleep well for several days in a roll such as going for boy scout camping, it could cause relapse.”

Psychiatrist 5: “Physical conditions like hyperthyroid, pregnancy, and other concurrent drugs for medical illness could trigger relapse.”

### Theme 5: Natural course of disease

Even though patients followed all treatment plans and lived their normal lives, there was still a chance of relapse due to a natural pattern of schizophrenia as addressed below.

Psychiatrist 2: “It’s about a natural course of disease. In some cases, relapses just happened even the patients adhered to treatment. Good or poor treatment outcome also depend on several factors.”

Psychiatric nurse 1: “Some patients took antipsychotic drugs regularly, but it still relapsed.

Psychologist 2: “Some patients had got relapse due to their uncooperativeness, but for some patients, relapse occurred in spite of good drug adherence.”

## Discussion

Thailand is a developing country with an upper-middle income level and a population of approximately 70 million in 2021. Healthcare for psychiatric patients in Thailand is mainly provided by government-owned hospitals. Khon Kaen Rajanagarindra Psychiatric Hospital is one of five psychiatric hospitals in Northeast Thailand responsible for four provincial health areas (Roi-Et, Khon Kaen, Maha Sarakham, and Kalasin). This qualitative study revealed multiple important factors associated with relapse in FES from perspectives of health professionals working at the Khon Kaen Rajanagarindra Psychiatric Hospital. These factors were grouped into five themes: drug adherence, family factors, substance abuse, concurrent health problems, and natural course of schizophrenia.

Drug adherence problems were further categorized into drug discontinuation, limited access to new generation drugs, self-dose reduction and skipping medication, and poor insight. Treatment guidelines for FES recommend at least one-year of antipsychotic drugs must be used following remission ^18^. The patients who discontinued antipsychotic drugs expressed more positive symptoms, relapses, alcohol and cannabis use, reduced insight; and poorer quality of life ^19^. These patients were more likely to live alone or live without family members involved in treatments. In this present study, the health professionals reported that their patients discontinued antipsychotic drugs without consultation. A major reason for discontinuation were intolerable side effects including extrapyramidal motor symptoms (difficult speaking and walking), drooling, and drowsiness ^20^. These side effects usually result from first-generation drugs (FGAs, typical antipsychotics), which predominantly block dopaminergic neurotransmission by inhibiting dopamine 2 and 3 receptors (D2R and D3R) in the brain ^21^. However, Liu-Seifert and colleagues reported different side effects i.e., weight gain, vomiting, dizziness, and fatigue in the patients using the second-generation antipsychotics (SGAs, atypical antipsychotics) ^22^. These drugs are dopamine-serotonin antagonists with a high affinity for serotonin 2A (5HT-2AR) and lesser affinity to 2C, 6, and 7 receptors (5HT-2CR, 5HT-6R, and 5HT-7R). The 5HT-2C antagonism contributes to the weight gain produced by some SGAs ^23^. Drug choice should be based on the patient’s clinical status and history of response to medication.

Some patients still followed a medication plan, but arbitrarily reduced the dose or skipped some medications. Novak and Švab conducted a semi-structured focus group qualitative study in schizophrenia patients and found that they skipped medication because of intolerable side effects ^24^. These side effects were reported to cause treatment-related stigma in the patients ^25^. In worse cases, patients had poor insight from deficits of metacognition (ability to think about thinking) ^26^. These patients lack insight into their own mental illness. As a result, they have poor adherence to treatment, subsequent illness relapse and rehospitalization. Verdoux and colleagues reported that over a period of first two years of FES, patients with poor medication adherence presented more frequently with an episodic course of illness and more readmission ^27^. Therapeutic programs that can improve medication adherence should be implemented early in the course of psychosis to reduce the deleterious consequences of poor medication adherence on clinical outcomes ^27^.

It has been reported that relapse rate in schizophrenia remains considerable even when prescribed medication adherence is well monitored ^28^. Therefore, it is intriguing to clarify what other factors influence this relapse. Family factors related to FES in the present study were stressful circumstances and family supports. Family is a key supporting unit for schizophrenia patients by encouraging them to find the way of coping with psychotic symptoms ^29^. In fact, family supports are important determinants for quality of life of schizophrenia patients ^30^. Loss of family members are related to functional changes in the brain. Previous study demonstrated reduction of rostral anterior cingulate cortex (rACC) activity in the brain of patients with complicated grief after loss events ^31^. It is known that the rACC is a region of the limbic system that is hypoactive during emotion processing in schizophrenia ^32^. This might explain why the loss event-induced stress can trigger relapse in FES.

As depicted in the interview summary, high expressed emotion (high EE) coincides with intense and negative verbal exchanges. This commonly results in oppositional and conflictual consequences ^33^. It has been revealed that living with family members with high EE doubled relapse risk ^34^. The high EE consists of five components: critical comments, hostility, emotional overinvolvement (EOI), positive remarks, and warmth ^35^. Critical comments are most common and exemplified by criticizing for being selfish or lazy, which are potential characteristics of negative symptoms ^36^. Hostility is manifested by general criticisms or attitudes that are rejecting of the patient ^35^. For example, caregivers state that patients are causes of problems and prefer to living away from them. Verma and colleagues found that applying psychoeducation program on problem-solving and communication skills in caregivers improved treatment outcomes in schizophrenia ^37^. In Bangkok, Thailand, Imkome and Waraassawapati reported that caregivers learned from their experiences how to control patient’s psychotic symptoms as well as recognizing warning signs of the symptoms ^38^. However, there are still no psychoeducational intervention or other methods applied to enhance their knowledge on mental health. In some countries, caregivers still believed that schizophrenia resulted from supernatural elements ^39^. From literature, improving the mental health literacy even among primary health care professionals is required in developing countries ^40^. These reports indicate that caregiver’s health literacy and attitude towards patients are still to be developed. Lastly, family’s poverty hinders psychotic symptom recovery and also limits resource allocation to family members, especially in low- and middle-income countries ^41, 42^. This present study indicated a positive influence of family supports on adaptation mechanisms of outpatients with schizophrenia. Hence, there is a need for relatives to provide support in order to facilitate adaptability.

Substance abuses are the third most frequently reported factors associated with relapse in schizophrenia ^43^. Alvarez-Jimenez and colleagues showed a three-fold increase in risk of relapse in FES patients with persistent substance uses ^8^. In this present study, illicit drugs such as amphetamine and methamphetamine; and psychoactive substances such as alcohol, energy drinks, coffee, and weight loss medications were reported as factors of psychotic relapse. Associated with higher frequency of relapse, several studies reported high comorbidity of substance abuse and schizophrenia ^44^. Almost 75% of the FES patients reported the use of at least one substance in their lifetime ^45^. Most frequently used substances are cannabis or alcohol alone and in combinations with opioids, stimulants, or cocaine ^46^. In addition, a qualitative study showed that individuals with schizophrenia used caffeine at higher rates than the general population ^47^. They consumed different types of caffeinated drinks the whole day including: cola, instant coffee, brewed coffee, tea, iced coffee, and energy drinks. In the same study, some participants also reported consumption of three or more different types of caffeinated drinks. In the present study, instant coffee drinks and energy drinks were major sources of caffeine. Most common reasons of substance use were for social recreation, for fun, and imitation of family members ^48^.

Exacerbation of psychotic symptoms in patients with schizophrenia is caused by the multi determinants (biology factors, psychological and social factors). Previous studies showed that various concurrent physical health conditions including sleep disturbances, thyroid dysfunctions, and pregnancy can worsen psychotic symptoms. Schizophrenia patients with sleep disturbances were at a greater risk for worsening of positive symptoms after antipsychotic discontinuation ^49, 50^. These are linked to various forms of psychopathology of serotonin and dopamine systems ^51^. Hyperthyroidism usually manifests with different psychiatric disorders such as anxiety, emotional psychosis, or depression ^52^. Meanwhile, hypothyroidism is associated with negative symptoms of schizophrenia ^52^. Sit and colleagues reported that postpartum psychosis occurred in 2/1,000 childbearing women within the first four weeks after delivery ^53^. Etiology of the postpartum psychosis is hormonal shift after delivery in women with a history of mental illness ^54^. Women with broadly defined affective psychoses were more likely to relapse earlier in the postpartum period ^55^. 

Some patients experience relapse due to their uncooperativeness, but some patients still manifest relapse in spite of regular drug adherence. Hui and co-workers reported that relapse rates for the patients discontinuing and continuing medication were 79% and 41%, respectively ^56^. In the discontinuation group, frontal dysfunction and dopamine hyperactivity predict relapse occurrence. Meanwhile in the continuation group, relapse is primed by signs of cognitive control deficits (cognitive disinhibition – inability to tune out stimuli that are irrelevant to the task/process) ^57^. The proper cognitive control depends on coordination of various parts of the brain e.g., the medial frontal cortex, parietal regions, and dorsolateral prefrontal cortex (DLPFC) ^58^. In schizophrenia patients with FES, it has been observed that levels of neurotransmitters (N-acetylaspartate and glutamate-glutamine-γ-aminobutyric acid complex) in the DLPFC are abnormal ^59^. Altogether, abnormality of the brain per se is another potential cause of relapse in the FES. 

## Conclusion

Health care professionals perceived non-adherence to antipsychotic medication as a major together with family-related problems, substance abuses, concurrent health problems, and nature of schizophrenia. Adherence to antipsychotic medication, family support, and refraining from addictive substances were viewed as protective factors. The results also suggested that strengthening mental health psychoeducation by mental health professionals might help reduce relapse. This study calls for improvement in mental health care service delivery to individuals with schizophrenia. Establishing a program in mental health care that aims to produce competent mental health caregivers and professionals would improve clinical outcomes in mental health care service delivery.

## Data availability

### Underlying data

OSF: Risk and protective factors of relapse in patients with first-episode schizophrenia from perspectives of health professionals: a qualitative study in northeastern Thailand. https://doi.org/10.17605/OSF.IO/4FHE5 ^14^.

This project includes the following underlying data.

- Raw data-Bilingual transcripts of interviews.rar

### Extended data

OSF: Risk and protective factors of relapse in patients with first-episode schizophrenia from perspectives of health professionals: a qualitative study in northeastern Thailand. https://doi.org/10.17605/OSF.IO/4FHE5 ^14^.

This project includes the following extended data.

- Appendix 1 (Qualitative research study profiles)

- Appendix 2 (Qualitative research study prior relationship/contact with research participants)

- Bilingual interview guide

- Coding tree analysis

- A copy of written informed consent form

### Reporting guidelines

OSF: COREQ checklist for ‘Risk and protective factors of relapse in patients with first-episode schizophrenia from perspectives of health professionals: a qualitative study in northeastern Thailand’. https://doi.org/10.17605/OSF.IO/4FHE5 ^14^.

Data are available under the terms of the Creative Commons Zero "No rights reserved" data waiver (CC0 1.0 Public domain dedication).
