# Supplementary material for: Risk and protective factors of relapse in patients with first-episode schizophrenia from perspectives of health professionals: a qualitative study in northeastern Thailand
Source: F1000Res. 2021 Jun 25;10:499. [Version 1] doi: 10.12688/f1000research.53317.1 (PMC9379331; doi:10.12688/f1000research.53317.1)
Supplement: Peer review history

# Peer review: Reviewer response for version 1

**DOI:** 10.5256/f1000research.56686.r145345

**Published:** 2022-08-15

**Authors:** Padmavati Ramachandran, Greeshma Mohan

**Affiliations:** 1 Department of Psychosocial Rehabilitation, Schizophrenia Research Foundation, Chennai, India; 2 Schizophrnia Research foundation, Chennai, Tamil Nadu, India

This paper aimed to identify risk and protective factors related to relapse in first-episode schizophrenia (FES) in northeastern Thailand from the perspectives of health professionals. A qualitative research design has been used. Results indicate multiple factors affect relapse rates. 

 A critical review indicates several observations:

1. Language needs to be checked throughout the paper for grammatical errors and also use of appropriate terms (e.g. use of the term 'schizophrenic' is not acceptable).
2. The abstract mentions content analysis whereas the data analysis section seems to describe thematic analysis. 
3. Introduction: Would impaired cognitive ability be a positive symptom?
4. Methods: How many FGD and IDI?
5. Results:
  a) Use one term to refer to participants - either 'medical staff' or 'health professionals'.
  b) Repetitive quotes which convey the same meaning can be avoided. Some themes appear to have too many verbatim quotes and this makes the paper too lengthy.
  c) It might be helpful to use the term 'medication' instead of 'drugs' in Theme 1. Subsequent themes involve substance use and use of the term 'medication' may avoid confusion.
  d) The sub theme family supports could be made more reader-friendly by having further sub-headings and highlighting the most relevant quotes only. There is too much information under the heading currently.
6. Discussion:
  a) The term 'expressed emotion' has been mentioned and this was not used in the results section.
  b) *Sit and colleagues reported that postpartum psychosis occurred in 2/1,000 childbearing women within the first four weeks after delivery - *This statement is not entirely accurate. Women with mental illness have a chance of relapsing within the first 4 weeks.

Is the work clearly and accurately presented and does it cite the current literature?

Yes

If applicable, is the statistical analysis and its interpretation appropriate?

Not applicable

Are all the source data underlying the results available to ensure full reproducibility?

Yes

Is the study design appropriate and is the work technically sound?

Yes

Are the conclusions drawn adequately supported by the results?

Yes

Are sufficient details of methods and analysis provided to allow replication by others?

Yes

Reviewer Expertise:

Schizophrenia research, physical health pf persons with medical comorbidity

We confirm that we have read this submission and believe that we have an appropriate level of expertise to confirm that it is of an acceptable scientific standard.

# Peer review: Reviewer response for version 1

**DOI:** 10.5256/f1000research.56686.r142224

**Published:** 2022-07-12

**Authors:** Soumitra Das

**Affiliations:** 1 Consultant Psychiatrist Emergency Mental Health Sunshine Hospital, NWMH, Melbourne, Australia

Even though it does not reflect anything new in psychiatry, the paper talks about Thai-specific issues. Understanding a doctor's perspective is more like knowing the clinical acumens instead of knowing the real issues. Such interviews can be replicated with the patients or family itself.

 The study is pretty easy and nothing much to add from my side. It's a straightforward study to do. I would recommend accepting it for indexing only because it brings area-specific data which might be useful for doctors in Thailand or similar countries.

 Overall the whole study might not have any value for the other parts of the globe.

Is the work clearly and accurately presented and does it cite the current literature?

Yes

If applicable, is the statistical analysis and its interpretation appropriate?

I cannot comment. A qualified statistician is required.

Are all the source data underlying the results available to ensure full reproducibility?

Yes

Is the study design appropriate and is the work technically sound?

Yes

Are the conclusions drawn adequately supported by the results?

Yes

Are sufficient details of methods and analysis provided to allow replication by others?

Yes

Reviewer Expertise:

Psychiatry, ADHD , Youth mental health, ECT

I confirm that I have read this submission and believe that I have an appropriate level of expertise to confirm that it is of an acceptable scientific standard.
